# Supplementary material for: Comprehensive Annotation and Functional Exploration of MicroRNAs in Lettuce
Source: Front Plant Sci. 2021 Dec 24;12:781836. doi: 10.3389/fpls.2021.781836 (PMC8739914; doi:10.3389/fpls.2021.781836)
Supplement: Supplementary file 2 [file Data_Sheet_8.ZIP › GSM2230750.plot/Lsa-miRN3_Lsat_1_v5_gn_1_6001.1_905_TPlot.pdf]

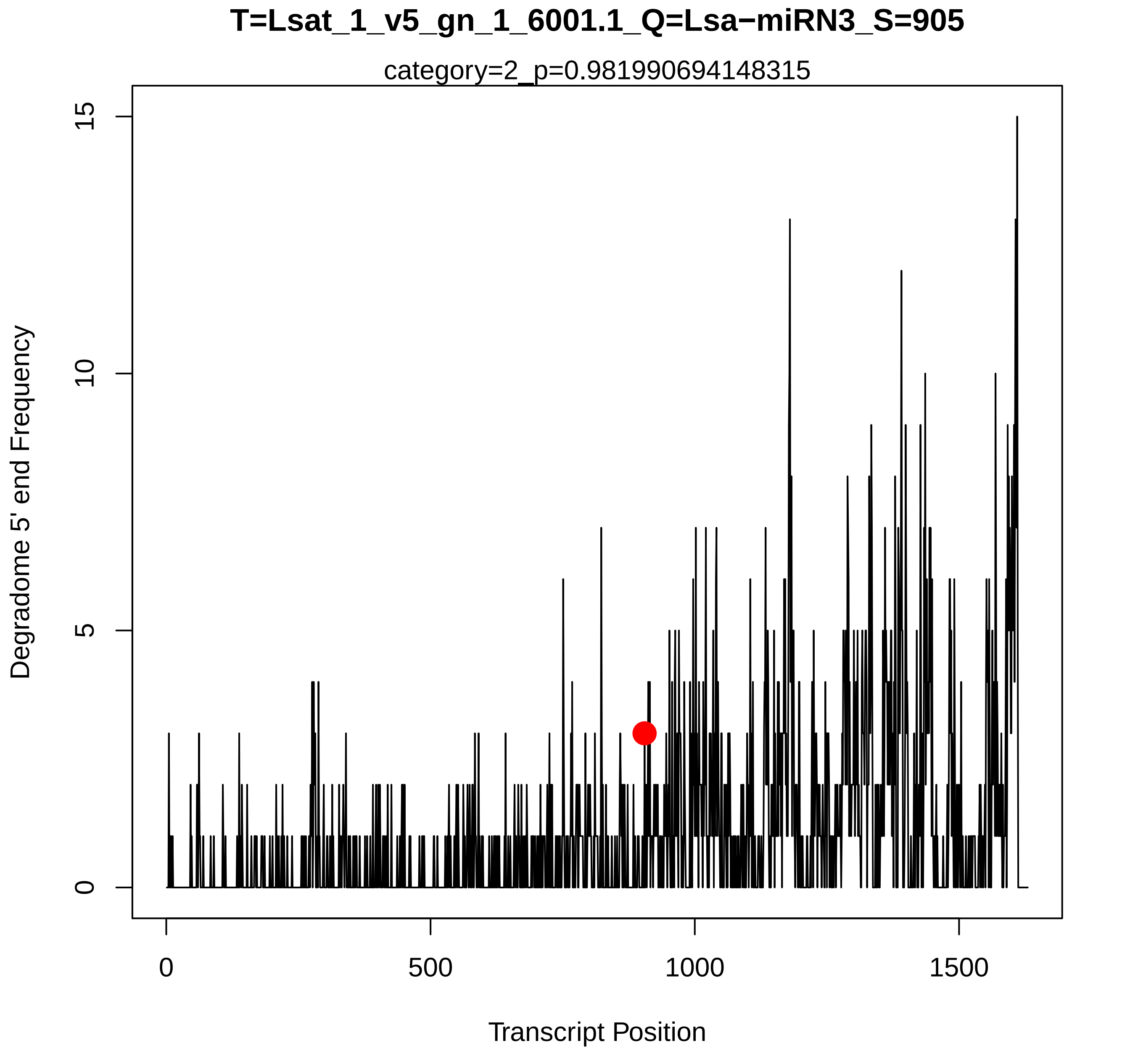

T=Lsat_1_v5_gn_1_6001.1_Q=Lsa−miRN3_S=905
category=2_p=0.981990694148315
Degradome 5' end Frequency
0
5
10
15
0
500
1000
1500
Transcript Position
